# Biosynthesis of Alkylcitric Acids in *Aspergillus niger* Involves Both Co-localized and Unlinked Genes

**DOI:** 10.3389/fmicb.2020.01378

**Published:** 2020-06-30

**Authors:** Sylvester Palys, Thi Thanh My Pham, Adrian Tsang

**Affiliations:** Centre for Structural and Functional Genomics, Concordia University, Montreal, QC, Canada

**Keywords:** alkylcitric acid, *Aspergillus niger*, secondary metabolite gene cluster, metabolite biosynthesis, phylogenomic analysis, transcription factor

## Abstract

Filamentous fungi are an abundant source of bioactive secondary metabolites (SMs). In many cases, the biosynthetic processes of SMs are not well understood. This work focuses on a group of SMs, the alkylcitric acids, each of which contains a saturated alkyl “tail,” and a citrate-derived “head.” We initially identified their biosynthetic gene cluster and the transcriptional regulator (*akcR*) involved in the biosynthesis of alkylcitrates in the filamentous fungus *Aspergillus niger* by examining the functional annotation of SM gene clusters predicted from genomic data. We overexpressed the transcription regulator gene *akcR* and obtained from one liter of culture filtrate 8.5 grams of extract, which are represented by seven alkylcitric acids as determined by NMR. Hexylaconitic acid A comprised 94.1% of the total production, and four of the seven identified alkylcitrates have not been reported previously. Analysis of orthologous alkylcitrate gene clusters in the Aspergilli revealed that in *A. oryzae* and *A. flavus* an in-cluster gene displays sequence similarity to *cis*-aconitate decarboxylase, the orthologue of which in *A. niger*, NRRL3_00504, is located on a different chromosome. Overexpression of the *A. niger* NRRL3_00504 and *akcR* genes together shifted the profile of alkylcitrates production from primarily hexylaconitic acids to mainly hexylitaconic acids, suggesting that NRRL3_00504 encodes an enzyme with hexyl aconitate decarboxylase activity. We also detected two additional, previously unreported, alkylcitric acids in the double overexpression strain. This study shows that phylogenomic analysis together with experimental manipulations can be used to reconstruct a more complete biosynthetic pathway in generating a broader spectrum of alkylcitric compounds. The approach adopted here has the potential of elucidating the complexity of other SM biosynthetic pathways in fungi.

## Introduction

Filamentous fungi are a rich source of secondary metabolites (SMs). Many fungal SMs have useful bioactive properties; for example, the antibiotic penicillin, the cholesterol-lowering drug lovastatin, and the anti-cancer compound griseofulvin (Fleming, 1929; Rubinstein et al., 1991; Panda et al., 2005). In fungi, genes involved in the biosynthesis of SMs are typically co-localized in the genome and they are referred to as SM gene clusters (Gacek and Strauss, 2012). These SM gene clusters generally consist of a backbone gene and multiple tailoring genes (Pi et al., 2015; Lind et al., 2017). The backbone genes encode enzymes including polyketide synthases, non-ribosomal peptide synthetases, polyketide/nonribosomal peptide hybrid enzymes, dimethylallyl tryptophan synthases, terpene cyclases, and fatty acid synthases. The backbone enzymes generate the core of a particular set of SM compounds, serving as a scaffold for further modifications by tailoring enzymes (Pi et al., 2015). Tailoring enzymes are thus responsible for most of the diversity of SMs (Lind et al., 2017) and can serve as potential targets of manipulation for diverting secondary metabolism away from or toward particular compounds (Urlacher and Girhard, 2012). Secondary metabolite gene clusters may contain a transporter gene which facilitates the export of SMs out of the cell (Pitkin et al., 1996; Desjardins and Proctor, 2007; Coleman and Mylonakis, 2009). Lastly, a SM gene cluster can also contain gene(s) encoding transcriptional regulator(s) which may facilitate the transcription of the entire cluster and start the process of SM biosynthesis (Fernandes et al., 1998). Overexpression of these in-cluster transcription factor genes can be a useful strategy to activate a SM gene cluster (Zabala et al., 2012).

Fungal orphan compounds are compounds that have been isolated from fungal cultures where the genes involved in their biosynthesis have yet to be determined. In *Aspergillus niger* and closely related black Aspergilli, approximately 140 orphan compounds have been isolated under laboratory conditions (Nielsen et al., 2009; Palys, 2017). These orphan compounds can potentially be linked to their biosynthetic gene clusters by inferring the type of enzymes that are likely to be involved in their production, then locating a cluster(s) with the genes that code for those enzymes. This strategy has been successfully applied to locate the gene cluster of kotanin from *A. niger* (Gil Girol et al., 2012) and phomoidride from *Taleromyces stiptitatus* (Fujii et al., 2015). Identifying the SM gene clusters involved in the biosynthesis of orphan compounds provides an approach to study their biosynthesis, enhance their production, and manipulate the pathway toward desired products.

Multiple approaches have been devised over the past decades to identify and produce fungal SMs (Fuchser et al., 1995; Helge et al., 2000). More recent approaches take advantage of the wealth of information collected through whole genome sequencing and genome annotation to accelerate novel compound discovery (Gil Girol et al., 2012; Zabala et al., 2012; Clevenger et al., 2017; Oakley et al., 2017). The accumulated body of genomic information from many different organisms has allowed for the bioinformatic prediction of SM gene clusters. The predicted gene clusters have revealed that many organisms have far more potential regarding SM production than what has already been discovered. For example, only 12% of the curated SM gene clusters of *A. niger* have experimental support (Inglis et al., 2013; Palys, 2017). In *Aspergillus nidulans* and *Aspergillus oryzae*, 13 and 3% of the predicted SM genes have experimental support, respectively, (Inglis et al., 2013; Palys, 2017).

**FIGURE 1:**
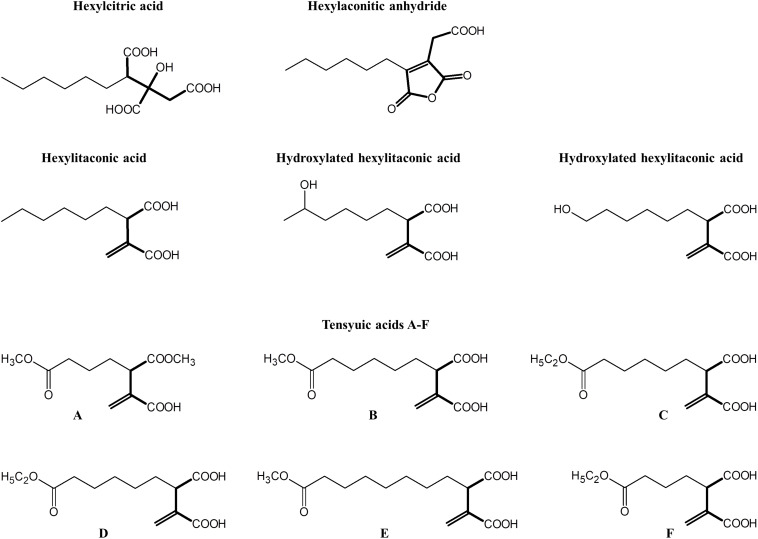
Structures of alkylcitric acids previously isolated from *A. niger* (Widenmuller et al., 1972; Almassi et al., 1994; Mondal et al., 2000; Hasegawa et al., 2007; Li et al., 2011). All these compounds show two common moieties: a saturated hydrocarbon “tail” (thin bonds) and a citrate-derived “head” (thick bonds).

Alkylcitrates comprise two moieties; a saturated alkyl “tail” and a “head” derived from citric acid. Alkylcitrates isolated from filamentous fungi include the tensyuic acids (A–F; Widenmuller et al., 1972; Hasegawa et al., 2007), hexylaconitic acid anhydride (Widenmuller et al., 1972; Mondal et al., 2000), hexylitaconic acid (Akira et al., 1984; Mondal et al., 2000), hexylcitric acid (Almassi et al., 1994), and hydroxylated hexylitaconic acids (Li et al., 2011; Figure 1). Members of alkylcitric acids have been shown to possess useful bioactive properties including plant root growth promotion (hexylitaconic acid; Mondal et al., 2000), anti-fungal (hexylaconitic acid anhydride; Koch et al., 2014), antibiotic, and anti-parasitic properties (tensyuic acid C; Hasegawa et al., 2007). The production level of these bioactive alkylcitrates under laboratory conditions is low (ng-mg/L range; Almassi et al., 1994; Mondal et al., 2000; Hasegawa et al., 2007; Li et al., 2011). In this work, we sought to use genomic information and chemical structure data to determine the SM gene cluster responsible for the production of the alkylcitric acid orphan compounds, manipulate their expression to increase the production of specific compounds, and to produce previously uncharacterized alkylcitric acids.

## Materials and Methods

### Alkylcitric Acid Gene Cluster Assignment and Orthologous Cluster Analysis

The alkycitric acid biosynthetic gene cluster was assigned based on the annotation of SM gene clusters in the *A. niger* NRRL3 genome available at the Genozymes website^1^. Orthologous alkylcitric acid clusters were located using the published Aspergilli genomes available at the Joint Genome Institute (JGI) MycoCosm website^2^. BLASTP queries were carried out to locate orthologous gene clusters using the sequences of the fatty acid synthase backbone enzymes (gene IDs NRRL3_11763 and NRRL3_11767) and the citrate synthase enzyme (NRRL3_11764) from the predicted *A. niger* NRRL3 alkylcitric acid cluster. Clusters were considered orthologous to the *A. niger* NRRL3 alkylcitric acid gene cluster when the following three criteria were met: (1) protein sequence identity based on BLASTP of individual encoding genes was >50% and query coverage was >50%; (2) the orthologous genes are co-localized; and (3) the orthologous gene cluster contained fatty acid synthase backbone genes as well as citrate synthase and citrate dehydratase genes.

### Strains, Culture Conditions, and Transformation Protocols

The *A. niger* strain PY11 (N593 *glaA::hisG*; Storms et al., 2005; Table 1) was used for production of alkylcitric acids. The DH5α strain of *Escherichia coli* was used for the propagation of cloned plasmids. Fungal cultures were initiated by inoculating spores (final concentration of 2 × 10^6^ spores/mL) into liquid minimal medium “J” (MMJ; Master et al., 2008) in 96-well microplate at 250 μL per well or in petri dishes [14 centimeter in diameter (Sarstedt), containing 120 mL per plate]. For growth of *A. niger* strains lacking a *pyrG* selection marker gene, MMJ media were supplemented with 10 mM uridine. For production of SMs, the cultures were incubated without shaking at 30°C for 5 days.

**TABLE 1:** Strains and plasmids used in the study.

**Strains**	**Genotype**	**Description**	**References**
PY11	N593 *glaA::hisG*	Δ*glaA* (glucoamylase) derivative of N593	Master et al., 2008
SP1	PY11 *akcR*^OE^	Overexpression of transcription regulator gene NRRL3_11765 (*akcR*) in PY11	This study
SP2	SP1 *hadA*^OE^	Overexpression of hexylaconitic acid decarboxylase gene NRRL3_00504 (*hadA*) in SP1	This study
SP3	SP1 Δ*NRRL3_11763-7*	Deletion of genes NRRL3_11763, NRRL3_11764, NRRL3_11765, NRRL3_11766, NRRL3_11767 in SP1	This study
**Plasmids**			
ANEp7		Extrachromosomal gene expression plasmid	Master et al., 2008
ANIp7		Integrative gene expression plasmid derived from ANEp7 by removing autonomously replicating sequence AMA1	Unpublished
ANIp9		Derived from ANIp7 by replacing the *pyrG* with an *amdS* selection marker	This study
ANIp7SP1		Derived from ANIp7 by inserting the coding region of the NRRL3_00504 gene between the glucoamylase promoter and terminator in ANIp7	This study
ANIp9SP2		Derived from ANIp9 by inserting the coding region of NRRL3_11765 gene between the glucoamylase promoter and terminator in ANIp9	This study
ANEp8-Cas9		Extrachromosomal *cas9* expressing plasmid	Sambrook and Russell, 2006
ANEp8-Cas9-gRNA- NRRL3_11764		Extrachromosomal *cas9* expressing plasmid with gRNA targeting the NRRL3_11764 locus	This study
			

Gene transformation in *Escherichia coli* was carried out by heat shock of competent cells (Master et al., 2008). For transformation of *A. niger*, spores were inoculated into liquid complete media (Song et al., 2018) and shaken at 250 rpm for 18 h prior to protoplasting. Protoplasting and transformation of *A. niger* were carried out as described (Master et al., 2008).

### Construction of Overexpression Cassettes for NRRL3_11765 and NRRL3_00504

The plasmids used in this study are listed in Table 1 while the primers used for PCR amplifications are listed in Supplementary Table S1. The plasmid ANIp9 was used to construct the vector for the overexpression of the fungal-specific transcription factor gene NRRL3_11765. To amplify NRRL3_11765, primers PR_1, and PR_2 that have gene-specific sequences and an additional 22 nucleotides of adapter sequence were used. The NRRL3_11765 gene was amplified from genomic DNA isolated from *A. niger* strain N593 (Sambrook and Russell, 2006), and purified with a GeneJET Genomic DNA Purification Kit (Thermo K0721, Thermo Scientific, Grand Island, NY, United States). The ANIp9 plasmid was amplified by PCR using primers Pr_3 and Pr_4 that contain an additional 22 nucleotides which are complementary to the adapter sequence of Pr_1 and Pr_2. The amplified vector and insert fragments were annealed and transformed into *E. coli* for propagation (Aslanidis and de Jong, 1990). The resulting plasmid was transformed into the *A. niger* strain PY11, creating strain SP1 that overexpresses NRRL3_11765. Positive colonies were screened based on their ability to grow on acetamide as the sole source of nitrogen (Kelly and Hynes, 1985).

The plasmid ANIp7 was used to construct the vector for the overexpression of NRRL3_00504, employing the same approach as for the NRRL3_11765 overexpression vector. The NRRL3_00504 gene was amplified using primers Pr_5 and Pr_6 while ANIp7 was amplified with Pr_3 and Pr_4. The resulting overexpression vector was transformed into strain SP1, creating strain SP2 which overexpresses NRRL3_00504 in the NRRL3_11765^OE^ background. Positive colonies showed growth on transformation plates containing no uridine.

### Construction of a Deletion Cassette for the Removal of Genes NRRL3_11763–NRRL3_11767

A linear DNA cassette was designed to delete five genes encompassing NRRL3_11763–NRRL3_11767 in the predicted alkylcitrate biosynthetic gene cluster. The deletion cassette contained 1255 base pairs (bp) of flanking DNA upstream of NRRL3_11763 (5’ flank) and 1331 bp of DNA downstream of NRRL3_11767 (3’ flank). Primers PR_7 and PR_8 were used to amplify the 5’ flank and primers PR_9 and PR_10 were used to amplify the 3’ flank. The 5’ and 3’ flanks were joined by overlap PCR (Yolov and Shabarova, 1990) using 22 nucleotide adapter sequences from primers PR_8 and PR_9 which are complementary to each other (Supplementary Figure S1). The deletion cassette was co-transformed into strain SP1 (NRRL3_11765^OE^) along with the CRISPR/Cas9 vector ANEp8-Cas9-gRNA-NRRL3_11764. The CRISPR/Cas9 system was co-transformed to induce a double stranded break in the NRRL3_11764 gene and facilitate homologous recombination of the deletion cassette (Song et al., 2018). Amplification of the gRNA insert for the CRISPR/Cas9 plasmid was carried out using primers PR_11 and PR_12. CRISPR/Cas9 plasmid was constructed as previously described (Song et al., 2018). Screening for the deletion was done by multiplex PCR with primers Pr_13 and Pr_14 that bind to the genome outside the 5’ and 3’ homologous regions, respectively, and primers Pr_15 and Pr_16 that bind to the NRRL3_11765 gene inside the deletion (Supplementary Figure S1). The expected band for the deletion strain is 2697 bp with primers Pr_13 and Pr_14, and no amplification of the NRRL3_11765 gene (Pr_15 and Pr_16). In the parental strain, the expected band for Pr_15 and Pr_16 is 1653 bp while for Pr_13 and Pr_14 no band is expected since the size of the amplicon is over 25 kb (Supplementary Figure S1).

### Sample Preparation and Data Analysis by Liquid Chromatography Mass Spectrometry

Following growth of the transformants and parental strain, 75 μL of growth cultures were collected in 1.5 mL microfuge tubes and centrifuged at 16,000 × *g* for 45 min to remove mycelia, spores and cellular debris. The supernates were transferred to new tubes and an equal volume of cold methanol (−20°C) was added for protein precipitation. Following incubation on ice for 10 min, samples were centrifuged at 16,000 × *g* for 45 min to remove precipitated proteins. Supernates were then transferred to fresh tubes and an equal volume of 0.1% formic acid was added.

Electrospray liquid chromatography mass spectrometry (LC-MS) analyses were performed on a 7-Tesla Finnigan LTQ-FT-ICR mass spectrometer (Thermo Electron Corporation, San Jose, CA, United States). Ionization voltage used was 4900 V in positive mode and 3700 V in negative mode. Scan range was from 100 to 1400 m/z at a 50,000-resolution setting. The solvent delivery system used was a Series 200 auto sampler and micropump (Perkin Elmer, Waltham, MA, United States). Injection volume was 10 μL and flow rate was 250 μL/min. Reversed-phase liquid chromatography separation was performed using an Eclipse C18 3.5 μm, 2.1 × 150 mm column (Agilent, Santa Clara, California, United States). The solvents used to generate the gradient during the separation were 0.1% formic acid in water for solvent A and 0.1% formic acid in acetonitrile for solvent B. The gradient was used to elute the metabolites: 5% B isocratic for 1 min, increased to 95% B in 10 min, isocratic at 95% B for 1 min, decreased to 5% B in 0.1 min, and isocratic at 5% B for 5.9 min.

### Sample Preparation and Data Analysis by Gas Chromatography Mass Spectrometry

For gas chromatography mass spectrometry (GC-MS) analysis, 0.1 mL of growth culture was extracted twice with 1 mL of ethyl acetate. The extract was dried under a stream of nitrogen. Dried extracts were then dissolved in 50 μL of N, O-bis(trimethylsilyl)trifluoroacetamide (BSTFA; Supelco, Sigma-Aldrich, St. Louis, Missouri, United States), followed by heating at 70°C for 30 min (Toussaint et al., 2012). The trimethysilyl derivatized product in BSTFA was then injected in a Hewlett Packard HP6890 gas chromatograph equipped with a HP5975 mass detector and a DB-5MS column (25 m × 0.2 mm; Santa Clara, California, United States). The program started at 80°C (held for 2 min), followed by an increase of 15°C per min to 315°C and held at 315°C for 3 min. The mass spectrometer was operated in the electron impact ionization mode.

### Secondary Metabolite Purification and Structural Analysis by Nuclear Magnetic Resonance Spectra

One liter of the growth cultures of strain SP1 (NRRL3_11765^OE^) were extracted with 1 L of ethyl acetate followed by two additional extractions with 500 mL of ethyl acetate each. The extract was dried *in vacuo* to yield a brown syrup. A portion of the material (850 mg) was dissolved in 2 mL of a 4:1 (v/v) mixture of acetonitrile and high performance liquid chromatography (HPLC)-grade water. The sample was injected (100 μL/injection) into an Atlantis dC18 OBD Prep Column (100 Å, 5 μm, and 19 mm × 100 mm; Waters, Milford, Massachusetts, United States) connected with an Agilent 1100 series HPLC at a flow rate of 20 mL/min. Solvent A was HPLC-grade water containing 0.1% formic acid and solvent B was acetonitrile. The HPLC gradient included a linear increase from 5% to 95% of solvent B over the course of 10 min, and then remain at 95% of B for 3 min. Solvent B was returned to 5% within 1 min and then held for 5 min to allow for column re-equilibration. The detector was set at a wavelength of 210 nm. Compounds were collected based on peak signals. The collected materials were re-extracted with ethyl acetate and dried to yield brown syrups ready for nuclear magnetic resonance (NMR) analysis.

Secondary metabolites from the strain SP2 (SP1, NRRL3_00504^OE^) were extracted and purified following the identical method used for strain SP1. These alkylcitric acids were eluted and collected from HPLC at different retention times.

For NMR analysis, the dried syrup materials were dissolved in deuterated chloroform or deuterated methanol (Supplementary Table S2). All NMR spectra were recorded with a Varian VNMRS-500 MHz) at 25°C.

## Results

### Locating the Biosynthetic Gene Cluster for the Alkylcitric Acids

**FIGURE 2:**
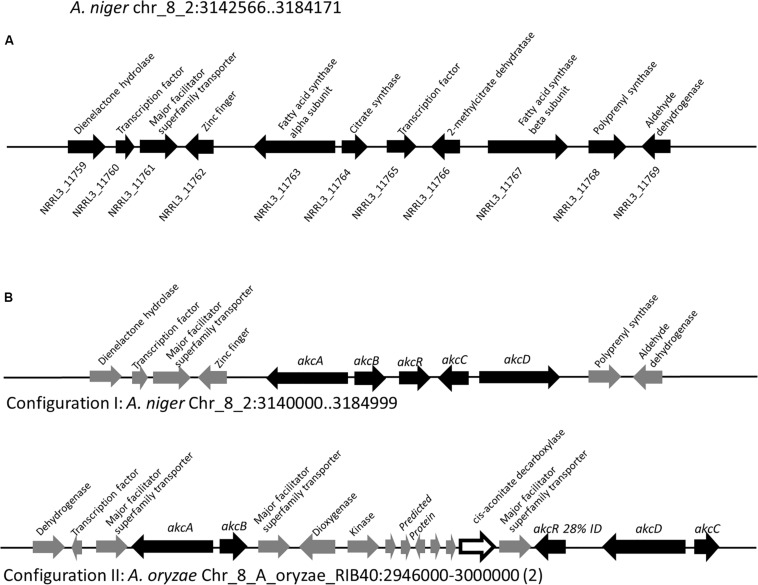
**(A)** Region of Chromosome VIII of *A. niger* containing the predicted alkylcitric acid gene cluster. Note that the boundaries of the gene cluster has not been defined. **(B)** Two configurations of the alkylcitric acid cluster in the Aspergilli. The percent identity (28%) displayed between *akcR* and the in-cluster transcription factor in *A. oryzae* represents a BLASTP top hit for *akcR*, but which is below the cut off (% identity > 50%) to be considered orthologous.

To locate the gene cluster involved in alkylcitric acid production, we began by examining the structures of the alkylcitric acids that have previously been reported (Figure 1). Their structures consist of a saturated hydrocarbon “tail” and a “head” derived from citric acid, suggesting that a fatty acid synthase or a highly reducing polyketide synthase is involved in the synthesis of the hydrocarbon “tail” while citrate generating and/or modifying enzyme(s) are involved in the production of the citrate moiety. Using the secondary metabolite gene clusters annotated in the genome of *A. niger* NRRL3, we located a single candidate gene cluster spanning genes NRRL3_11759-NRRL3_11769 (Figure 2A). The cluster contains genes encoding a dienelactone hydrolase (NRRL3_11759), two fungal-specific transcription factors (NRRL3_11760 and NRRL3_11765), transporter (NRRL3_11761), fatty acid synthase alpha and beta subunits (NRRL3_11763 and NRRL3_11767), a citrate synthase (NRRL3_11764), a 2-methylcitrate dehydratase (NRRL3_11766), a polyprenyl synthase (NRRL3_11768), and an aldehyde dehydrogenase (NRRL3_11769).

### Overexpression of Transcription Factor Gene NRRL3_11765 Leads to the Overproduction of Secondary Metabolites

The gene NRRL3_11765 encodes a protein that contains a Zn(2)-C6 fungal-type DNA-binding domain (PF00172) and a fungal-specific transcription factor domain (PF04082). As such, it was annotated to encode a fungal-specific transcription factor. Since the gene NRRL3_11765 is located in the predicted alkylcitric acid gene cluster, it is predicted to be the transcription regulator of the alkycitrate biosynthesis genes. To overexpress NRRL3_11765, we placed the gene under the control of the maltose-inducible glucoamylase promoter (Ganzlin and Rinas, 2008). The recombinant gene was introduced into *A. niger* for random integration into the genome. Following growth in the presence of the inducer maltose, we analyzed the extracellular medium of strain SP1 (NRRL3_11765^OE^) and the parental PY11 strain by GC-MS. Strain SP1 showed multiple peaks while extracellular medium of the parental strain did not show any of these peaks (Supplementary Figure S2).

### Nuclear Magnetic Resonance (NMR) Analysis Reveals Seven Alkylcitric Acids

**FIGURE 3:**
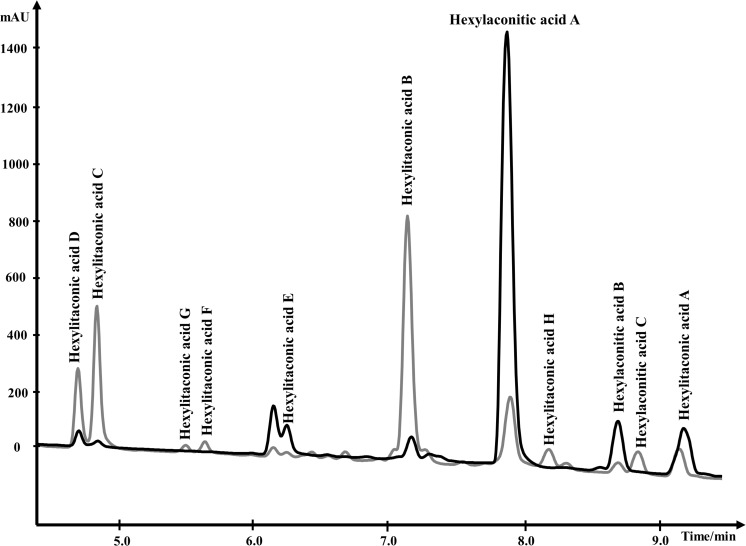
Overlapping HPLC chromatograms of SMs produced by strain SP1 (*akcR*^OE^), in black, and by strain SP2 (*akcR^OE^hadA^OE^*), in gray. Structures of the compounds are shown in Figure 4.

For NMR analysis, extracellular culture fluid of strain SP1 was extracted with ethyl acetate and dried. About 8.5 grams of oily material were obtained from 1 L of extracellular fluid. Compounds in the material were separated by HPLC and were eluted from 4.0 min to 10.0 min (Figure 3). To elucidate the structure of SMs, purified compounds from HPLC were subjected to NMR and mass spectrometry analyses.

**FIGURE 4:**
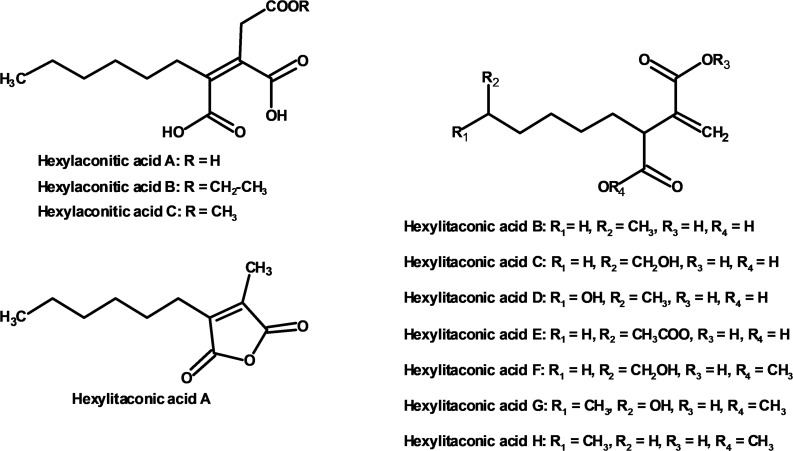
Compounds identified from growth media of strain SP1 (NRRL3_11765^OE^; hexylaconitic acids A and B, and hexylitaconic acids A to E) and of strain SP2 (*akcR^OE^ hadA^OE^*; hexylaconitic acid C and hexylitaconic acids F to H).

We used one-dimensional and two-dimensional NMR spectra to elucidate the structure of the two major metabolites produced by strain SP1: hexylaconitic acid and hexylitaconic acid (Supplementary Information, Section on structural elucidation). Structures of the other compounds were determined by comparing their ^1^H- and ^13^C-NMR spectra with the major hexylaconitic acid and hexylitaconic acid, and with published data (Almassi et al., 1994; Mondal et al., 2000; Klemke et al., 2003; Li et al., 2011). Since all compounds in the extract are derivatives of either hexylaconitic acid or hexylitaconic acid, we designate the major compounds as hexylaconitic acid A and hexylitaconic acid A. Their derivatives are then denoted with successive letters alphabetically. Seven compounds were identified from growth media of strain SP1 (NRRL3_11765^OE^), including hexylaconitic acids A and B, and hexylitaconic acids A to E. Compound structures are shown in Figure 4. Four of the seven compounds (hexylaconitic acids A, B, and hexylitaconic acids A, E) have not been reported before. The major compound hexylaconitic acid A (8 grams) comprised 94.1% of the crude extract (8.5 grams), hexylitaconic acid A comprised 1.8% (0.15 gram), with the remaining ∼4% for all the other five alkylcitric acid compounds combined. The NMR data of the seven alkylcitric acids are presented in Supplementary Table S2. Since all the compounds identified in strain SP1 (NRRL3_11765^OE^) are alkylcitric acids, we conclude that NRRL3_11765 encodes the regulator of alkylcitric acids biosynthesis, and call this gene *akcR*.

### Deletion of Genes NRRL3_11763 to NRRL3_11767 Abolishes the Production of Alkylcitric Acids

To confirm the involvement of the predicted alkylcitric acid cluster in the production of alkylcitric acids, we designed a deletion cassette to remove five co-localized genes from the cluster, NRRL3_11763 to NRRL3_11767. These genes code for fatty acid synthase subunits A and B (NRRL3_11763 and NRRL3_11767), citrate synthase (NRRL3_11764), the transcription regulator *akcR* (NRRL3_11765), and 2-methylcitrate dehydratase (NRRL3_11766). The deletion was constructed in strain SP1 (*akcR*^OE^) background where the *akcR* overexpression cassette is randomly integrated in the genome and remains active. Mass chromatogram of the cluster deletion strain did not contain peaks corresponding to any of the alkylcitric acids (Supplementary Figure S2). Given the lack of alkylcitric acid production in strain SP3 (*akcR*^OE^ ΔNRRL3_11763-7), we refer to this region of the genome as the alkylcitric acid gene cluster.

### Analysis of Orthologous Alkylcitric Acid Gene Clusters Reveals the Involvement of an Aconitate Decarboxylase

About 95% of the alkylcitric acids produced by strain SP1 were hexylaconitic acids and 5% were hexylitaconic acids (Figure 3). In the itaconic acid biosynthesis pathway, *cis-*aconitate decarboxylate converts aconitic acid to itaconic acid (Bentley and Thiessen, 1957; Hossain et al., 2016). Hence we posited that the conversion of hexylaconitic acid to hexylitaconic acid requires an enzyme with aconitate decarboxylase activity. To identify the candidate aconitate decarboxylase, we examined orthologous gene clusters predicted to be involved in alkylcitric acids biosynthesis. Previous reports have shown that alkylcitric acids and the similar structured maleidrides are being produced not only in *A. niger* but also in other filamentous fungi including *Penicillium striatosporum* (Stewart et al., 2005; Li et al., 2011) and *A. oryzae* (Wasil et al., 2018). Therefore, we used the alkylcitric acid cluster of *A. niger* as query to search for orthologous gene clusters in the published Aspergilli genomes. Twenty orthologous alkylcitric acid gene clusters are found in section Nigri and two in section Flavi (*A. flavus* and *A. oryzae*) of the Aspergilli (Supplementary Figure S3). The orthologous clusters are not identical (Figure 2B). They share five genes in common, which we call: *akcA*, fatty acid synthase alpha subunit (NRRL3_11763); *akcB*, citrate synthase (NRRL3_11764); *akcR*, transcription regulator (NRRL3_11765); *akcC*, 2-methylcitrate dehydratase (NRRL3_11766); and *akcD*, fatty acid synthase beta subunit (NRRL3_11767).

There were two configurations for the predicted alkylcitric acid gene clusters (Figure 2B). The first is found in section Nigri, as exemplified in *A. niger* NRRL3, contains the five conserved genes (conserved cluster) in a contiguous arrangement. The second is found in section Flavi, as exemplified in *A. oryzae*, where there are intervening genes within the conserved clusters.

Based on sequence similarity a gene predicted to encode *cis*-aconitate decarboxylase is in the alkylcitrate gene cluster of the genomes of the *A. flavus* and *A. oryzae*. The *A. niger* orthologue of this aconitate decarboxylase is NRRL3_00504 on Chromosome I, outside the alkylcitric acid cluster which is on Chromosome VIII. This gene may potentially be involved in alkylcitric acid biosynthesis.

### Overexpression of NRRL3_00504 Gene in *akcR*^OE^ Strain Shifts Production From Hexylaconitic Acid A to Hexylitaconic Acids

In the SP1 (*akcR*^OE^) strain, hexylaconitic acid A constitutes 94.1% of total extract with its derived products including hexylitaconic acids comprising less than 6%. This observation suggests that the abundance of hexylaconitic acid produced by the SP1 (*akcR*^OE^) strain is the result of a biosynthetic bottleneck. As in the itaconic acid biosynthesis pathway (Bentley and Thiessen, 1957; Hossain et al., 2016), we hypothesized that an aconitate decarboxylase mediates the conversion of hexylaconitic acids to hexylitaconic acids. The gene NRRL3_00504 is predicted to encode an aconitate decarboxylase and it is the closest orthologue to the predicted aconitate decarboxylase genes found in the alkylcitrate gene clusters of *A. oryzae* and *A. flavus*. To examine the involvement of NRRL3_00504 in the biosynthesis of hexylitaconic acids, we overexpressed NRRL3_00504 in strain SP1 (*akcR*^OE^). We obtained ∼8 g/L of metabolites in strain SP2 (*akcR*^OE^ NRRL3_00504^OE^). However, in strain SP2 ∼10% of the metabolites are hexylaconitic acid A and 64% of the metabolites are hexylitaconic acids (B, C, and D; Figure 3). Since the shift of production of hexylaconitic acids to hexylitaconic acids is mediated by NRRL3_00504, the result provides experimental evidence that NRRL3_00504 encodes a hexylaconitic acid decarboxylase. We therefore name NRRL3_00504 as *hadA*.

**FIGURE 5:**
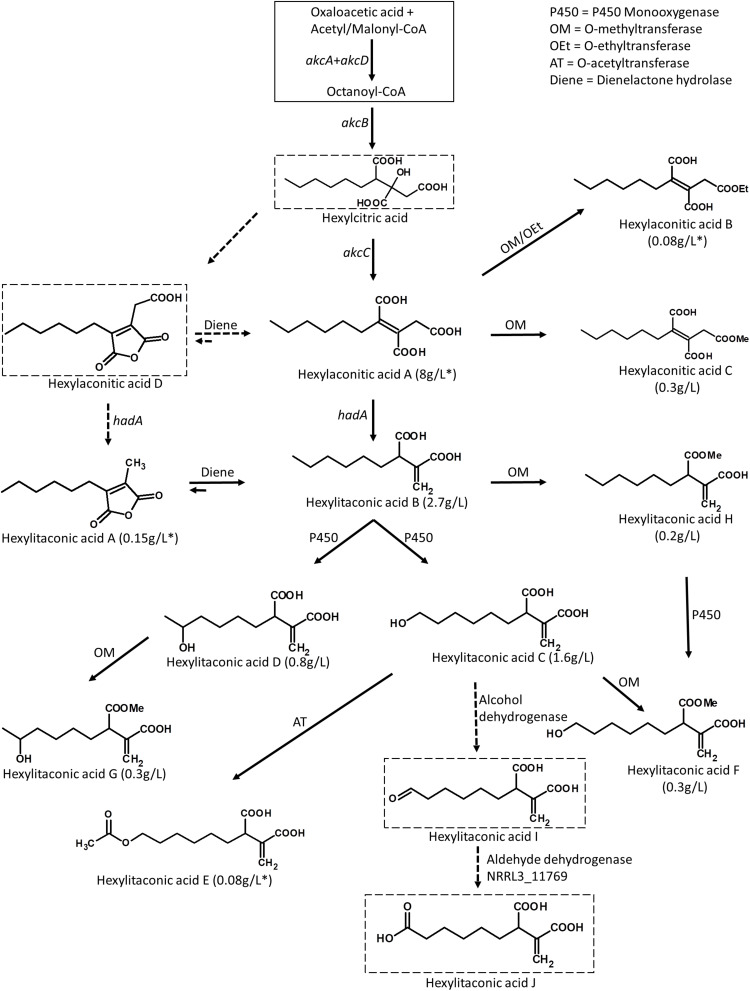
Predicted biosynthetic pathway for the alkylcitric acids. The solid box represents building blocks of hexylcitric acid, which were not detected in this work. Dashed boxes indicate compounds which were detected by mass spectrometry; all other compounds were purified and identified by NMR. Solid arrows indicate steps for which the products were purified and their structures resolved by NMR. Dashed arrows indicate steps for which the products were determined with MS by comparison to published compounds. Yields indicated with an asterisk (*) represent amounts obtained from the SP1 (*akcR*^OE^) strain; all other amounts represent yields obtained from the SP2 (*akcR^OE^ hadA^OE^*) strain.

Further, strain SP2 (*akcR^OE^hadA^OE^*) produces four additional alkylcitric acids: hexylaconitic acid C and hexylitaconic acids F to H (Figure 3). Two of these four compounds (hexylaconitic acid C and hexylitaconic acid F) have not been reported before. Each of these compounds was produced at ∼0.2–0.3 g/L range (Figure 5). The NMR data for these compounds are shown in Supplementary Table S2.

## Dicussion

Overexpression of the transcription factor *akcR* and the hexylaconitic acid decarboxylase gene *hadA* led to the production of 11 alkylcitric acids. Based on the chemical structure of the alkylcitric acids resolved by NMR and the genes co-localized in the genome of *A. niger* and related species in section Nigri, we hypothesize that the biosynthetic pathway for alkylcitric acids involves at least six co-localized genes and one unlinked gene: *akcA* and *akcD* (NRRL3_11763 and NRRL3_11767) for the two subunits of fatty acid synthase, *akcB* (NRRL3_11764) for citrate synthase, *akcC* (NRRL3_11766) for 2-methylcitrate dehydratase, NRRL3_11759 for dienelactone hydrolase, NRRL3_11769 for aldehyde dehydrogenase, and the unlinked *hadA* (NRRL3_00504) for hexylaconitic acid decarboxylase. The reconstructed pathway is shown in Figure 5.

The pathway is predicted to begin with the production of hexylcitric acid, which is generated by a citrate synthase (*akcB*) and fatty acid synthase (*akcA* and *akcD*). Despite finding peaks in LC-MS data [(M+H)^+^ = 277.1287; Supplementary Figure S4], we were unable to isolate and confirm the structure of hexylcitric acid by NMR. This initial compound may be efficiently converted to downstream hexylaconitic acid A and only present in small quantities. The next step in the pathway is the dehydroxylation of hexylcitric acid by 2-methylcitrate dehydratase (*akcC*) to produce hexylaconitic acid A. Finally, a decarboxylation reaction occurs to yield hexylitaconic acid B. This step was carried out by hexylaconitic acid decarboxylase (*hadA*) whose role was confirmed in this study.

The alkylcitrate pathway shares similarity with the itaconic acid pathway from *A. terreus* (Bentley and Thiessen, 1957; Hossain et al., 2016), since they share a similar chemical structure and gene annotations. The itaconic acid pathway starts by the production of citric acid from acetyl-CoA and oxaloacetic acid followed by a dehydroxylation to aconitic acid and finally a decarboxylation to itaconic acid. These steps are carried out by a citrate synthase, a dehydratase and a *cis-*aconitate decarboxylase, respectively, (Bentley and Thiessen, 1957; Hossain et al., 2016). The biosynthetic pathway of itaconic acid and the proposed pathways for hexylitaconic acid B are shown in Supplementary Figure S5.

The next steps of the alkylcitrate biosynthetic pathway may involve an omega oxidation reaction. This three-step reaction (Vanhanen et al., 2000) would require one or more P450 monooxygenase(s) to generate hydroxyl groups (hexylitaconic acids C and D). An alcohol dehydrogenase then oxidizes the hydroxyl group to generate an aldehyde (hexylitaconic acid I). Finally an aldehyde dehydrogenase is required to generate a carboxyl group (hexylitaconic acid J; Vanhanen et al., 2000). Although we could not confirm the structures of hexylitaconic acid I and J by NMR, we detected their molecular masses [(M+H)^+^ = 229.1076 and (M+H)^+^ = 245.1025, respectively] by LC-MS (Supplementary Figure S4). While we do not know the locations in the genome for the P450 monooxygenase(s) and the alcohol dehydrogenase, an aldehyde dehydrogenase (NRRL3_11769) is co-localized with the alkylcitric acid gene cluster (Figure 2A). As in the case of *hadA*, the omega oxidation step and perhaps other biosynthetic reactions in the alkylcitric acid pathway appear to be generated by enzymes whose genes do not co-localize. We do note, however, that not all clustered genes have been completely ruled out as candidates for these steps.

Other genes which co-localize with the alkylcitric acid cluster may also be involved in alkylcitric acid biosynthesis. In particular, the clustered gene NRRL3_11759 encoding a dienelactone hydrolase, an anhydride ring-opening enzyme (Schlömann et al., 1993), may be responsible for shifting the ringed hexylaconitic acid D and hexylitaconic acid A toward the non-ringed hexylaconitic acid A and hexylitaconic acid B (Figure 5). This would provide an explanation for why the non-ringed forms were more abundant than the ringed forms. For instance, the non-ringed form (hexylaconitic acid A) was detected as major compound in the SP1 (*akcR*^OE^) extract (∼95% of total extract) while its ringed form (hexylaconitic acid D) was detected by LC-MS [(M+H)^+^ = 241.1076; Supplementary Figure S4] but not by purification process, indicating a lower level of production. Similarly, hexylitaconic acid B was the major compound (∼34% of total extract) of the SP2 (*akcR^OE^ hadA^OE^*) strain while its ringed form (hexylitaconic acid A) was isolated at ∼1.5% of the total extract.

In addition to the hexyl “tail” modifications, we also detected one O-ethylated (hexylaconitic acid B), one O-acetylated (hexylitaconic acid E), and four O-methylated (hexylaconitic acid C and hexylitaconic acids F to H) alkylcitric acids indicating other enzymes capable of appending the hydroxyl and carboxyl groups of the alkylcitric acids may also be involved in the pathway. No such transferase genes are located nearby or inside the alkylcitric acid cluster and we are also unable to locate them in orthologous clusters in other filamentous fungal species.

There are six previously published alkylcitric acids (tensyuic acids A–F; Figure 1) that we did not detect from our strains (SP1 and SP2). Tensyuic acids B to D appear to be o-methylated and o-ethylated forms of hexylitaconic acid J. The low production of hexylitaconic acid J and its direct precursor (hexylitaconic acid I) would provide an explanation for the absence of tensyuic acid B to D in our samples, despite identifying other o-methylated and o-ethylated compounds of the pathway. Locating and overexpressing the responsible alcohol dehydrogenase and aldehyde dehydrogenase genes should therefore help to increase the production of tensyuic acid B to D. Tensyuic acids A and F are products of o-methylation and o-ethylation of the 4-carbon-tail itaconic acid while tensyuic acid E is an o-methylated form of the 8-carbon-tail itaconic acid. There is no published evidence supporting the ability of one fatty acid synthase to synthesize compounds containing different carbon chain lengths. We therefore predicted that tensyuic acids A, E, and F were synthesized by different fatty acid synthases, which are active in the *A. niger* FKI-2342 strain [tensyuic acid producer (Hasegawa et al., 2007)], but not in our strains (SP1 and SP2).

The common perception of fungal secondary metabolism in fungi has been that natural products are generated from discrete clusters of genes (Walton, 2000; Harvey et al., 2018; Robey et al., 2018). As our understanding increases, we begin to see more exceptions where SM genes are not co-localized. For example, the genes involved in melanin production are clustered in *A. fumigatus*, while in *A. niger* the genes are scattered throughout the genome (Chiang et al., 2011). The involvement of both co-localized and unlinked genes in SM biosynthesis has implications for heterologous expression as well as pathway reconstruction. Experimental design that focuses solely on clustered genes (Bojja et al., 2004; Szewczyk et al., 2008; Chiang et al., 2009, 2013; Geib and Brock, 2017) may fail to realize the full diversity of SM generation or result in partial pathway reconstructions. Analyzing orthologous clusters can help address some of these shortcomings by identifying unlinked genes that are involved in SM biosynthesis.

The overexpression strains SP1 (*akcR*^OE^) and SP2 (*akcR^OE^ hadA^OE^*) generated alkylcitric acids at the g/L level (defined here as artificial production) compared favorably to the levels of production previously reported from *A. niger* and other species (isolated without genetic manipulation and defined here as natural production; Petersen et al., 2015). For example, natural production of hexylitaconic acid B yielded 0.5 mg/L in *Penicillium striatisporum* (Li et al., 2011), 14 mg/L in *A. niger* K88 (Akira et al., 1984), and 14 mg/L in *A. niger* AN27 (Mondal et al., 2000). In our study, artificial production of hexylitaconic acid B yielded 214 mg/L in strain SP1 and increased to 2.7 g/L in strain SP2. This was 200 fold higher than that from natural production in other strains of *A. niger* and more than 5000 fold higher than *P. striatisporum* under natural production. Hexylaconitic acid A was produced at 8 g/L by the SP1 (*akcR*^OE^) strain. The compound was not detected in any previous study, however, its ringed, anhydride form (hexylaconitic acid D) was naturally produced at 19.84 mg/L from *A. niger* AN27 (Mondal et al., 2000). Compared to the hexylaconitic acid A titre in our study, this represents an increase of more than 400 fold. The increase of production of alkylcitric acids in this study is dramatic, given that SMs normally require years of research to reach similar titres (Zhang et al., 2002; Mulder et al., 2015).

One reason for this steep increase from natural and artificial production may be due to the upregulation of genes involved in the production of building blocks of the fatty acids (acetyl-CoA and malonyl-CoA; Hopwood and Sherman, 1990) and the citric acid and citric acid derived moieties (oxaloacetic acid). These building blocks can be generated from the breakdown of citric acid due to the action of a citrate lyase or similar enzyme (Chen et al., 2014) which may be upregulated following the upregulation of *akcR*. Since *A. niger* is a well-known producer of citric acid, a high yield of the alkylcitric acids was expected. In this context, an even higher production of alkylcitric acids may be achieved if the *akcR* is overexpressed in an industrial citric acid producing strain of *A. niger*.

## Conclusion

Our study has shown that using a combination of bioinformatics and chemical structure, orphan compounds can be traced back to their biosynthetic gene cluster. Activation of these gene clusters may lead to the overproduction of orphan compounds as well as the production of novel derivatives. Moreover, modification of the expression level of tailoring genes may be able to shift the biosynthetic pathway toward desired products. This strategy was applied to locate the biosynthetic gene cluster of the alkylcitric acids. Overexpressing the co-localized transcription factor *akcR* and an unlinked tailoring gene (*hadA*), we overproduced alkylcitric acids and obtained six previously unreported alkylcitric acids.

## Data Availability Statement

The raw data supporting the conclusions of this article will be made available by the authors, without undue reservation, to any qualified researcher.

## Author Contributions

SP performed the *in silico* analyses. TP carried out the purification and structure determination by mass spectra and nuclear magnetic resonance. SP and TP constructed the strains and performed mass spectrometry analyses. AT developed the project and provided intellectual inputs. All the authors wrote the manuscript and approved the final version of the manuscript.

## Conflict of Interest

The authors declare that the research was conducted in the absence of any commercial or financial relationships that could be construed as a potential conflict of interest.

## Footnotes

1 www.fungalgenomics.ca

2 https://genome.jgi.doe.gov/programs/fungi/index.jsf
