# Supplementary material for: Biosynthesis of Alkylcitric Acids in Aspergillus niger Involves Both Co-localized and Unlinked Genes
Source: Front Microbiol. 2020 Jun 30;11:1378. doi: 10.3389/fmicb.2020.01378 (PMC7338620; doi:10.3389/fmicb.2020.01378)
Supplement: Supplementary file 1 [file Data_Sheet_1.pdf]

Supplementary material

Table S1. Primers used in this study.

Primer Name	Description	Sequence 5' --> 3'
PR_1	NRRL3_11765 forward primer	TACTTCCAATCCAATCCATTTG ACGATATGGCTACTCCCCGCGCATC
PR_2	NRRL3_11765 reverse primer	TTATCCACTTCCAATCCATTTG TCACTGCATCAATCCCAACGCAG
PR_3	ANIp7 and ANIp9 reverse primer	AAATGGATTGGATTGGAAGTAC TGATCTAGCGTGTAATG
PR_4	ANIp7 and ANIp9 forward primer	AAATGGATTGGAAGTGGATAAC TTAATTAAGTTTAAACG
PR_5	NRRL3_00504 forward primer	TACTTCCAATCCAATCCATTTG ATGGTCACTATCACCGCCAAATC
PR_6	NRRL3_00504 reverse primer	TTATCCACTTCCAATCCATTTG CTACTGCAACGGATTGACCAC
PR_7	Cluster deletion 5' Flank forward primer	AAGCTTTCCTCTCAAGAGAC
PR_8	Cluster deletion 5' Flank reverse primer	AAATGGATTGGAAGTGGATAAC CATACGAGTTATATCACTCTGC
PR_9	Cluster deletion 3' Flank forward primer	TTATCCACTTCCAATCCATTTC GTCTGACGATTCGTCTGTG
PR_10	Cluster deletion 3' Flank reverse primer	GTTGCACTACTTTGAACCG
PR_11	CRISPR guide RNA NRRL3_11764 forward primer	TCTACTGCCACCATACCGGA GTTTTAGAGCTAGAAATAGCAAG
PR_12	CRISPR guide RNA NRRL3_11764 reverse primer	TCCGGTATGGTGGCAGTAGA GACGAGCTTACTCGTTTCG
PR_13	Screening of cluster deletion forward primer	CACGTACACAAACTCTACTGAG
PR_14	Screening of cluster deletion reverse primer	CATTGCCTTTGCTTTGCCTTAC
PR_15	NRRL3_11765 integration forward primer	GCAGTCGTCTGGGCAAGATTG
PR_16	NRRL3_11765 integration Re	CACTTCCAGGAAGGGGATGAG

*Overlapping sequences are underlined

Table S2: The NMR data for hexylaconitic acid A to C and hexylitaconic acid A to H

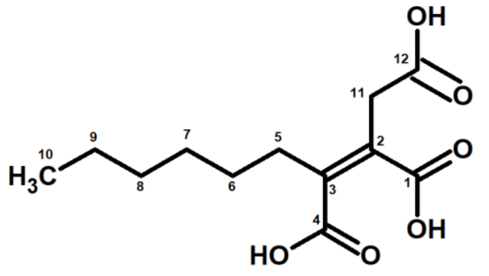

Hexylaconitic acid A

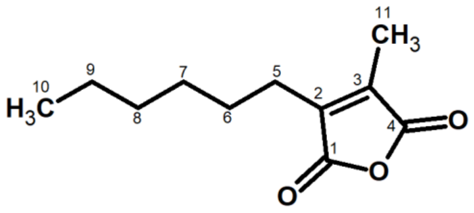

Hexylitaconic acid B

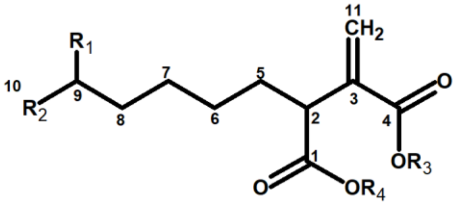

Hexylitaconic acids B to H

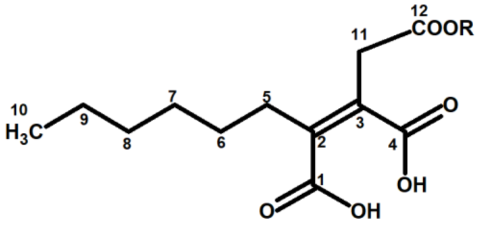

Hexylaconitic acids B and C

Hexylitaconic acid A: R_1= H, R_2 = CH_3, R_3 = H, R_4 = H

Hexylitaconic acid C: R_1 = H, R_2 = CH_2OH, R_3 = H, R_4 = H

Hexylitaconic acid D: R_1 = OH, R_2 = CH_3, R_3 = H, R_4 = H

Hexylitaconic acid E: R_1 = H, R_2 = CH_3COOCH_2, R_3 = H, R_4 = H

Hexylitaconic acid F: R_1 = H, R_2 = CH_2OH, R_3 = H, R_4 = CH_3

Hexylitaconic acid G: R_1 = OH, R_2 = CH_3, R_3 = H, R_4 = CH_3

Hexylitaconic acid H: R_1= H, R_2 = CH_3, R_3 = H, R_4 = CH_3

Hexylaconitic acid B: R = CH_2-CH_3

Hexylaconitic acid C: R = CH_3

	**Hexylaconitic acid A ($CDCl_3$)**		**Hexylaconitic acid B ($CDCl_3$)**		**Hexylaconitic acid C ($CDCl_3$)**	
	**^{13}C**	**^{1}H**	**^{13}C**	**^{1}H**	**^{13}C**	**^{1}H**
1	165.093		165.314		165.231	
2	147.950		147.503		147.632	
3	135.596		136.355		136.158	
4	165.07		165.185		165.155	
5	24.782	2.491, t (8.0)	24.877	2.483, t (7.5)	24.892	2.476, t (8.0)
6	27.309	1.596, m	27.495	1.592, m	27.48	1.586, m
7	28.971	1.283 ÷ 1.303, m	29.135	1.256 ÷ 1.323, m	29.135	1.251 ÷ 1.317, m
8	31.149	1.283 ÷ 1.303, m	31.297	1.256 ÷ 1.323, m	31.29	1.251 ÷ 1.317, m
9	22.24	1.283 ÷ 1.303, m	22.411	1.256 ÷ 1.323, m	22.411	1.251 ÷ 1.317, m
10	13.718	0.882, t (7.0)	13.957	0.889, t (7.0)	13.949	0.885, t (7.0)
11	29.1	3.566, s	29.582	3.504, s	29.286	3.513, s
12	172.56		167.211		167.666	
O-CH_3					52.849	3.748, s

O-**CH$_2$**-CH$_3$			62.054	4.205, q (7.0)		
O-CH$_2$-**CH$_3$**			14.033	1.271, t (6.0)		

	**Hexylitaconic acid A (CDCl$_3$)**		**Hexylitaconic acid B (CDCl$_3$)**		**Hexylitaconic acid C (CD$_3$OD)**	
	**^{13}C**	**^{1}H**	**^{13}C**	**^{1}H**	**^{13}C**	**^{1}H**
1	165.859		179.571		175.709	
2	140.415		46.288	3.485, t (7.0)	46.561	3.451, t (7.0)
3	144.755		136.909		139.428	
4	166.253		171.649		168.105	
5	24.426	2.451, dt (7.0; 0.5)	30.565	5a: 1.904, m<br>5b: 1.706, m	30.769	5a: 1.852, m<br>5b: 1.684, m
6	27.537	1.574, m	27.279	1.368 ÷ 1.245, m	27.241	1.323 ÷ 1.369, m
7	29.077	1.304, m	28.888	1.368 ÷ 1.245, m	28.857	1.323 ÷ 1.369, m
8	31.331	1.304, m	31.521	1.368 ÷ 1.245, m	25.328	1.323 ÷ 1.369, m
9	22.438	1.304, m	22.529	1.368 ÷ 1.245, m	32.113	1.518, m
10	13.969	0.889, t (7.0)	13.992	0.874, t (7.0)	61.556	3.532, t (7.0)
11	9.477	2.069, s	130.231	11a: 6.550, s<br>11b: 5.910, s	125.625	11a: 6.329, s<br>11b: 5.757, s

	**Hexylitaconic acid D (CD$_3$OD)**		**Hexylitaconic acid E (CDCl$_3$)**		**Hexylitaconic acid F (CD$_3$OD)**	
	**^{13}C**	**^{1}H**	**^{13}C**	**^{1}H**	**^{13}C**	**^{1}H**
1	175.703		179.156		174.246	
2	46.642	3.453, t (8.0)	46.877	3.148, t (7.0)	46.596	3.529, t (7.0)
3	139.49		137.296		139.015	
4	168.137		171.461		167.864	
5	30.812	5a: 1.857, m<br>5b: 1.691, m	29.688	5a: 1.928, m<br>5b: 1.735, m	30.622	5a: 1.857, m<br>5b: 1.679, m
6	27.321	1.318 ÷ 1.459, m	27.192	1.349, m	27.109	1.306 ÷ 1.364, m
7	25.196	1.318 ÷ 1.459, m	28.884	1.349, m	28.801	1.306 ÷ 1.364, m
8	38.545	1.318 ÷ 1.459, m	25.666	1.349, m	25.295	1.306 ÷ 1.364, m
9	67.063	3.695, m	28.476	1.607, m	32.11	1.514, m
10	22.055	1.133, d (7.0)	64.551	4.043 t (7.0)	61.501	3.529, t (7.0)

11	125.587	11a: 6.327, s<br>11b: 5.756, s	129.609	11a: 6.529, s<br>11b: 5.837, s	125.762	11a: 6.322, s<br>11b: 5.741, s
O-CH_3			171.355 (CH_3-**C**=O)		50.96	3.648, s
			20.992 (**CH_3**-C=O)	2.043, s		

	**Hexylitaconic acid G (CD_3OD)**		**Hexylitaconic acid H ($CDCl_3$)**	
	**^{13}C**	**^{1}H**	**^{13}C**	**^{1}H**
1	174.246		173.783	
2	46.619	3.477, t (7.0)	46.202	3.488, t (7.0)
3	139.315		137.812	
4	167.955		171.142	
5	30.683	5a: 1.880, m<br>5b: 1.690, m	31.214	5a: 1.886, m<br>5b: 1.676, m
6	27.207	1.125 ÷ 1.436, m	27.389	1.298 ÷ 1.210, m
7	25.151	1.125 ÷ 1.436, m	28.945	1.298 ÷ 1.210, m
8	38.522	1.125 ÷ 1.436, m	31.57	1.298 ÷ 1.210, m
9	67.002	3.693, m	22.54	1.298 ÷ 1.210, m
10	22.039	1.131, d (7.0)	14.01	0.869, t (7.0)
11	125.678	11a: 6.315, s<br>11b: 5.735, s	129.063	11a: 6.496, s<br>11b: 5.862, s
O-CH_3	50.96	3.649, s	52.075	3.682, s

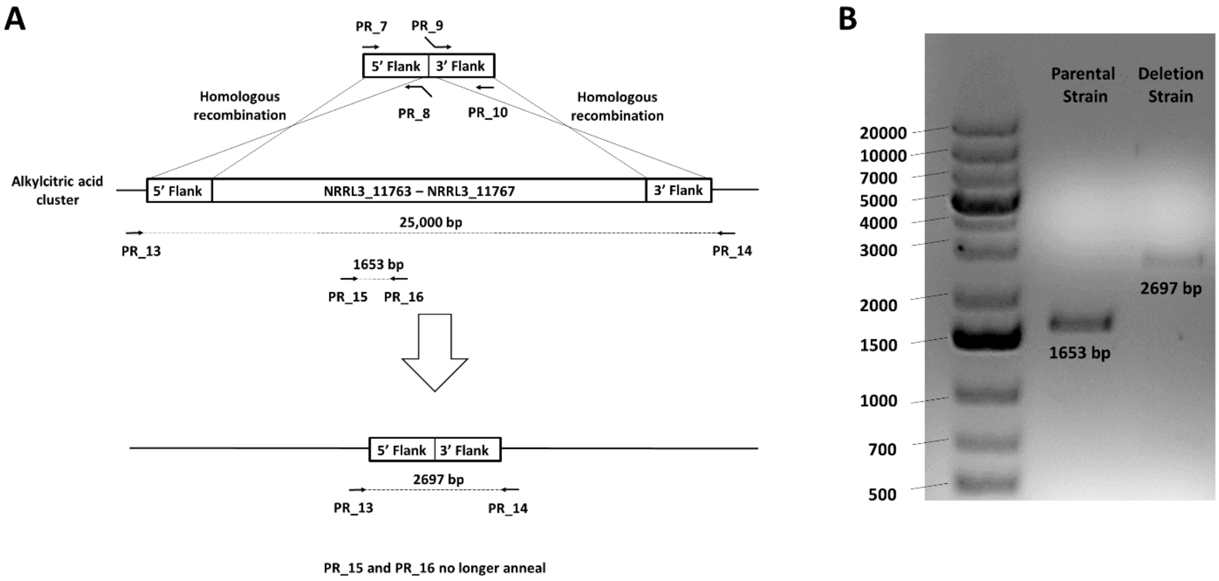

Figure S1. (A) Diagram showing the deletion of genes NRRL_11763-7 by homologous recombination. Primers used are indicated by arrows and are listed in Table S1. (B) Analysis by PCR confirming of the deletion of the alkylcitric acid cluster (ΔNRRL3_11763-7) in the SP1 (NRRL3_11765^{OE}) strain.

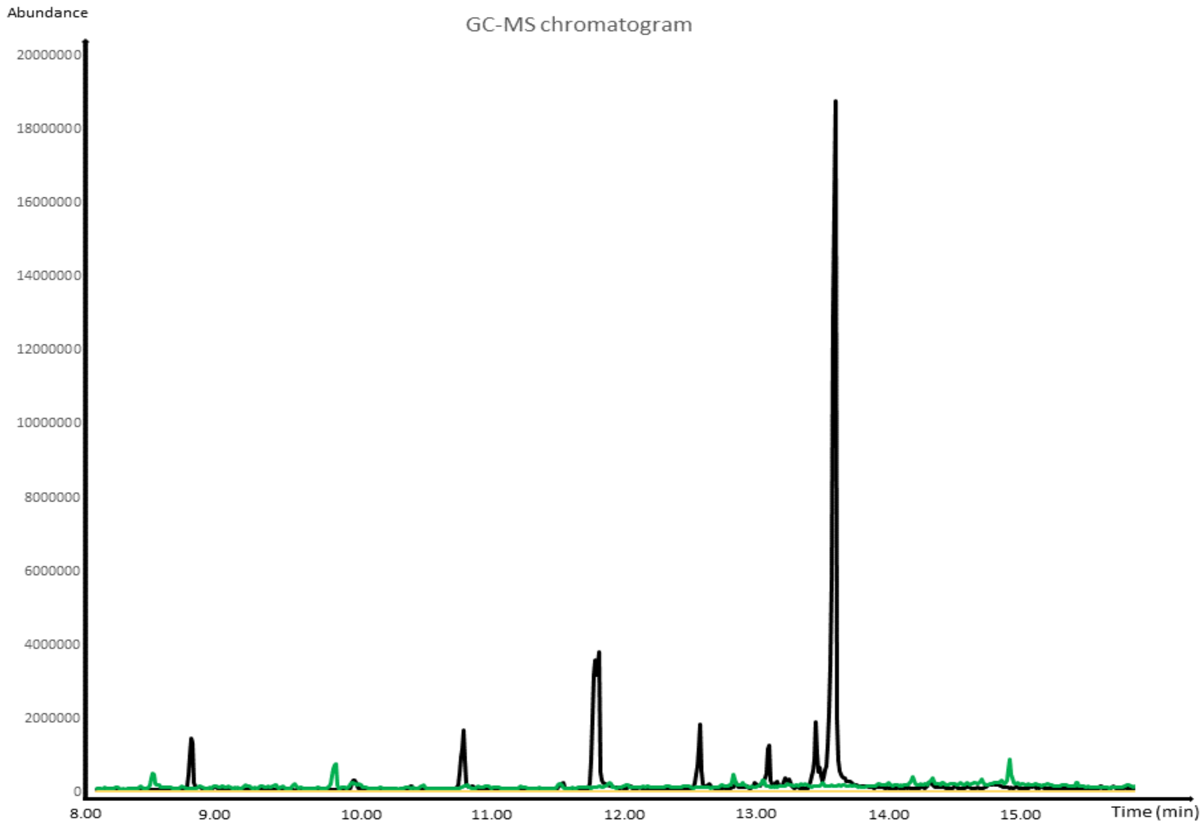

Figure S2. The GC-MS total ion chromatograms of the SP1 (*akcR*^{OE}) (in black) overlapping with parental strain *A. niger* PY 11 (in yellow), and with the SP3 (*akcR*^{OE} ΔNRRL3_11763-11767) strain (in green).

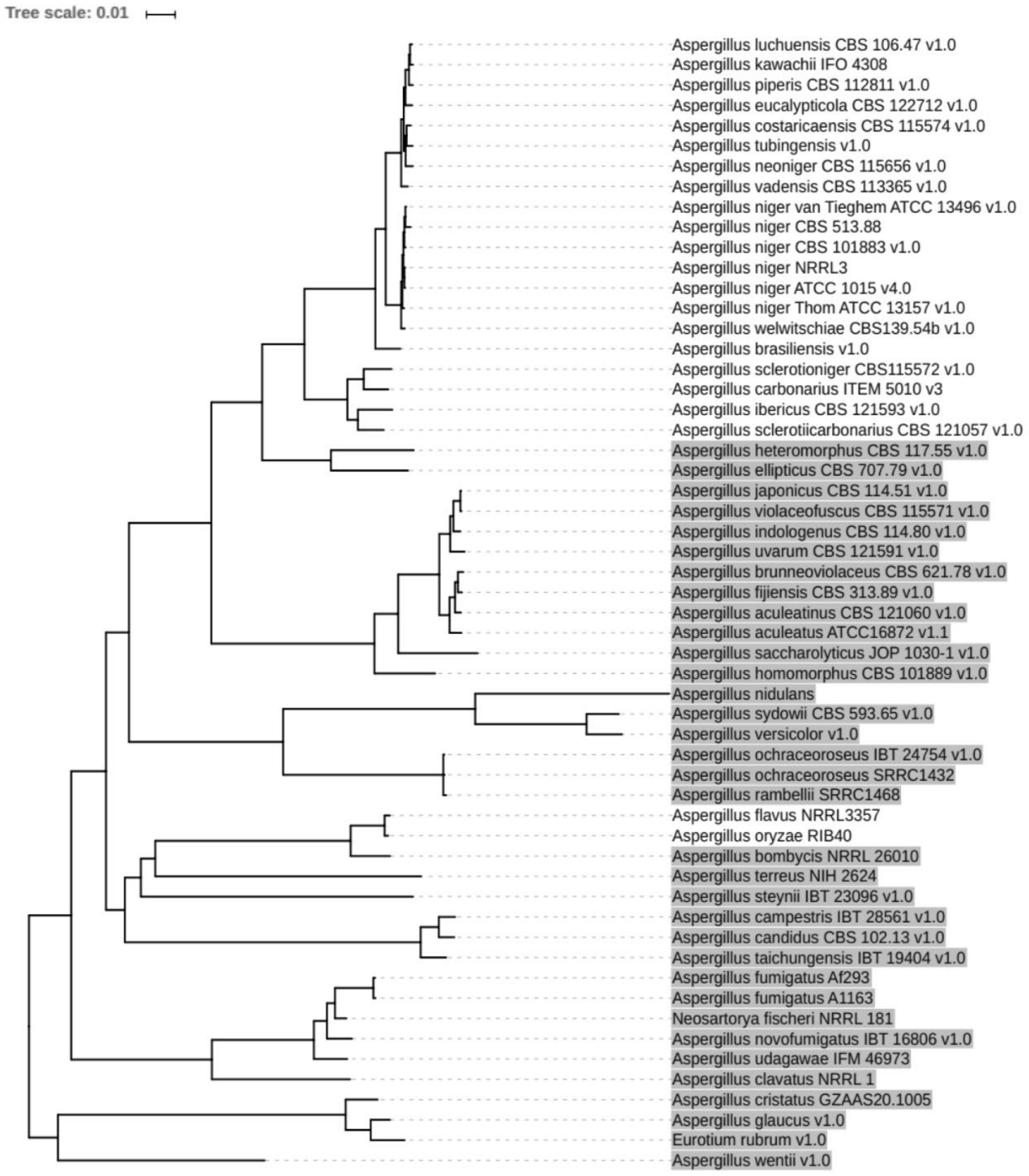

Figure S3: Taxonomic tree of published Eurotiomycetes genomes (https://genome.jgi.doe.gov/programs/fungi/index.jsf). Twenty orthologous alkylcitric acid gene clusters of section Nigri and two of section Flavi (*A. flavus* and *A. oryzae*) are displayed as non-highlighted names.

Structural elucidation process of hexylaconitic acid A and hexylitaconic acid B

Hexylaconitic acid A has an accurate mass (m/z) of 259.1176 ± 1.3 mmu ($[M+H]^+$) which indicates its molecular formula is $C_{12}H_{18}O_6$. According to ^{13}C-NMR and DEPT 135 spectra, compound 1 contains 12 carbons including 5 quaternary, 6 methylene, and 1 methyl group. Of the 5 quaternary carbons, 3 are of a carbonyl (δC 173.319, 165.138, 165.115) and 2 are of an alkene (δC 148.102, 135.490). Since there is no ^{13}C signal in the 40 - 130 ppm region, this suggests that there is no methylene or methyl group that is directly bonded to an oxygen atom. Therefore, the 3 carbonyl groups are defined as 3 carboxylic acid groups which is consistent with the presence of 6 oxygen atoms in the molecular formula. Five methylene groups (C5-C9) were shown by COSY-NMR and HMBC-NMR spectra to be chained together forming a saturated hydrocarbon tail ($-C_6H_{13}$) which ends at a methyl group (C10). The $-C_6H_{13}$ structural element is also confirmed by the splitting pattern of these protons. This partial structure was extended by observing the correlations between the saturated hydrocarbon chain, the carbonyl group (C4) and 2 alkene groups (C2, C3) on HMBC. According to ^{1}H-NMR, the last methylene group (C11) has a singlet signal (δH 3.549, 2H) which indicates it is separated from the other 5 methylene groups. The HMBC-NMR spectrum determined that this $-CH_2-$ group has correlations with 2 carbonyl groups (C1, C12) and 2 alkene groups (C2, C3) but has no correlation with the $–C_6H_{13}$ chain. On the basis of the previously mentioned NMR evidences, the structure was determined to be hexylaconitic acid A. The ^{1}H and ^{13}C-NMR chemical shifts are listed in Table S2. The selected COSY and HMBC correlations are shown in Figure S4.

Hexylitaconic acid A has molecular formula of $C_{12}H_{16}O_3$ and an accurate mass of m/z 209.1183 ± 1.0 mmu ($[M+H]^+$). The ^{13}C-NMR and DEPT 135 spectra indicate that compound 2 contains 12 carbons including 4 quaternary, 5 methylene, and 2 methyl groups. The ^{1}H and ^{13}C-NMR chemical shifts reveal that compound 2 also contains a saturated hydrocarbon chain ($-C_6H_{13}$) and it is identical to that of compound 1. The rest of the structure consists of 4 quaternary carbons and 1 methyl group. Three of these 4 quaternary carbons (C2, C3, C4; δC 144.755, 140.415, 165.859, respectively) have HMBC correlations with the methylene group (C5, δC 24.426) of the hydrocarbon chain. The methyl group has a singlet signal (δH 2.069, 3H) on the ^{1}H-NMR spectrum which is evidence that there is no correlation between itself and nearby protons. Moreover, according to the HMBC spectrum, this methyl group has correlations with 3

quaternary carbons (C2, C3, C1; δC 144.755, 140.415, 166.253, respectively). On the basis of the NMR evidences and its molecular formula, the only structure that is suitable was hexylitaconic acid A. Selected HMBC correlations are shown below and ^{1}H and ^{13}C NMR chemical shifts are listed in Table S2.

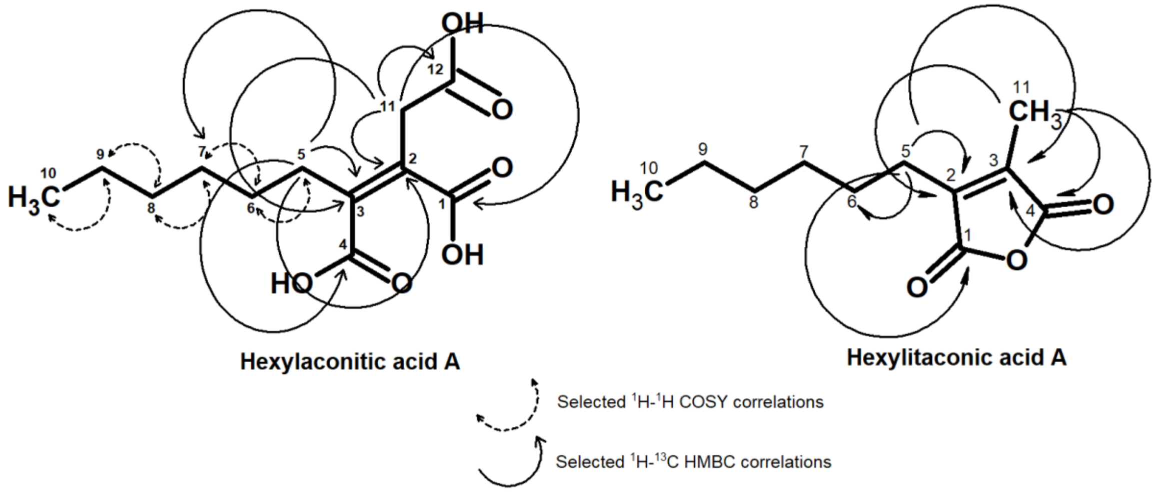

Structure and selected HMBC and COSY correlations of hexylaconitic acid A and hexylitaconic acid A

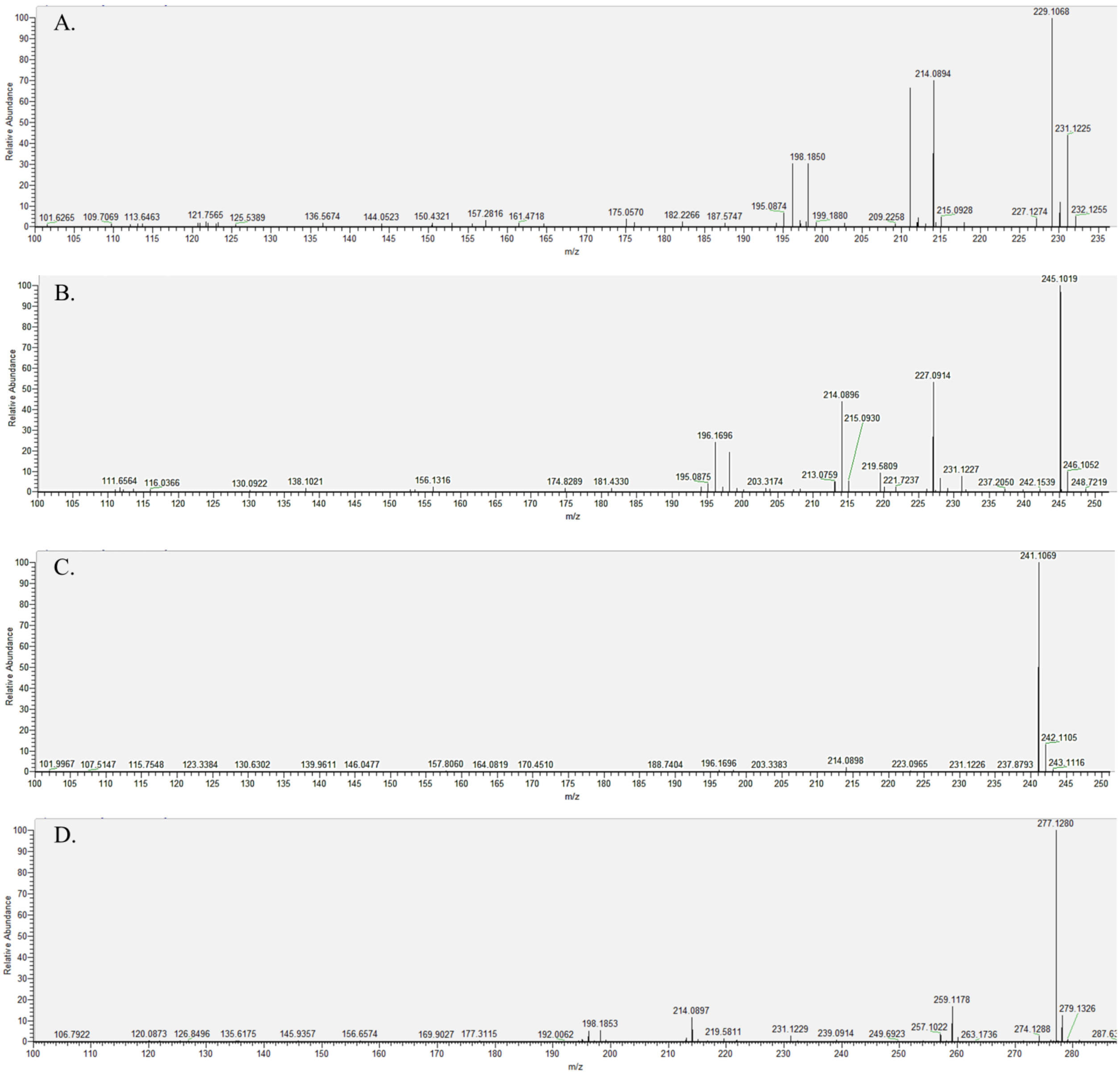

Figure S4. Mass spectra (LC-MS) of (A) hexylitaconic acid I ($[M+H]^+$ = 229.1076 ± 1.1 mmu), (B) hexylitaconic acid J ($[M+H]^+$ = 245.1025 ± 1.2 mmu), (C) hexylaconitic acid D ($[M+H]^+$ = 241.1076 ± 1.2 mmu) and (D) hexylcitric acid ($[M+H]^+$ = 277.1287 ± 1.4 mmu).

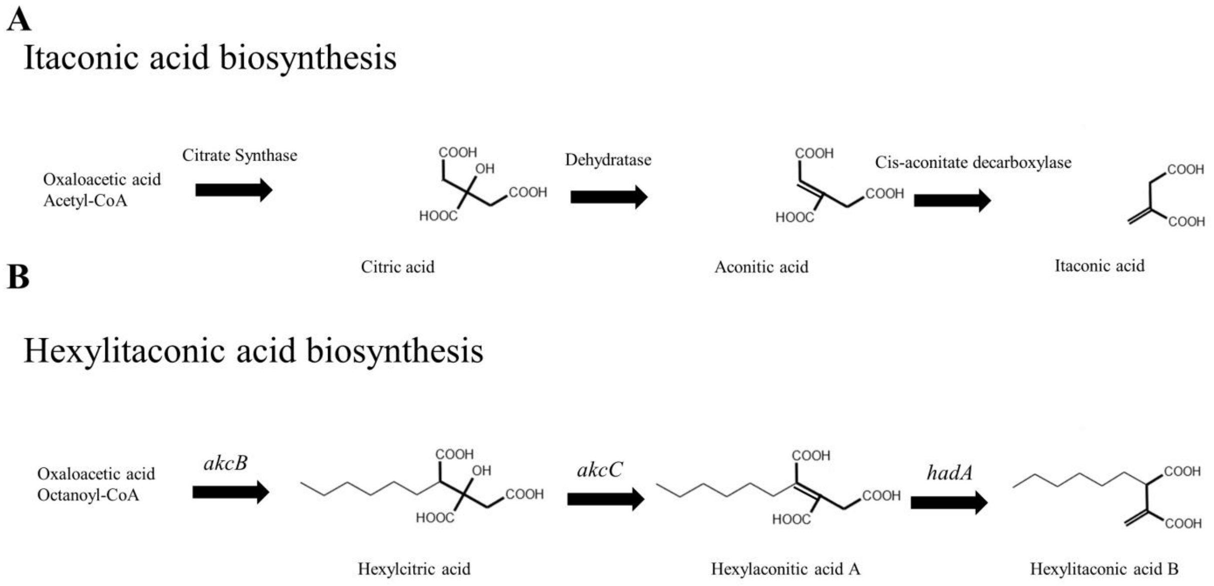

Figure S5. (A) Biosynthetic pathways of itaconic acid[39-40]. (B) The predicted hexylitaconic acid B pathway. Thick chemical bonds indicate the common moieties between the two pathways.
